# Basal and starvation-induced autophagy mediates parasite survival during intraerythrocytic stages of *Plasmodium falciparum*

**DOI:** 10.1038/s41420-018-0107-9

**Published:** 2018-10-03

**Authors:** Shiny Joy, Lavanya Thirunavukkarasu, Palak Agrawal, Archana Singh, B. K. Chandrasekhar Sagar, Ravi Manjithaya, Namita Surolia

**Affiliations:** 1 Molecular Biology and Genetics Unit, Jawaharlal Nehru Centre for Advanced Scientific Research, Jakkur, Bangalore, 560064 India; 2 CSIR-Institue of Genomics and Integrative Biology, Room 223, Sukhdev Vihar, Mathura Road, New Delhi, 110025 India; 3 Electron Microscopy Laboratory, Department of Neuropathology, National Institute of Mental Health and Neuro Sciences, Bangalore, 560029 Karnataka India

**Keywords:** Macroautophagy, Mechanisms of disease

## Abstract

The precise role of autophagy in *P. falciparum* remains largely unknown. Although a limited number of autophagy genes have been identified in this apicomplexan, only *Pf*Atg8 has been characterized to a certain extent. On the basis of the expression levels of *Pf*Atg8 and the putative *Pf*Atg5, we report that the basal autophagy in this parasite is quite robust and mediates not only the intraerythrocytic development but also fresh invasion of red blood cells (RBCs) in the subsequent cycles. We demonstrate that the basal autophagy responds to both inducers and inhibitors of autophagy. In addition, the parasite survival upon starvation is temporally governed by the autophagy status. Brief periods of starvation, which induces autophagy, help survival while prolonged starvation decreases autophagy leading to stalled parasite growth and reduced invasion. Thus, starvation-induced autophagy is context dependent. Importantly, we report characterization of another autophagy marker in this parasite, the putative *Pf*Atg5 (*Pf*3D7_1430400). *PfAtg*5 is expressed in all the intraerythrocytic stages and partially colocalizes with ER, mitochondria, apicoplast and *Pf*Atg8. It is also present on the double membrane bound vesicles. Altogether, these studies pave way for the detailed dissection of *P. falciparum* autophagy machinery and insights into molecular and functional characterization of its players for developing new therapeutics as antimalarials.

## Introduction

Macroautophagy (hereafter autophagy) in eukaryotes is a conserved catabolic process, whereby cytosolic constituents such as proteins and organelles are captured in double membrane vesicles called autophagosomes and are transported to lysosomes for degradation. The amino acids and other macromolecular constituents thus generated in lysosomes are recycled to maintain cellular homeostasis^1^. Autophagy thus serves as a response mechanism for survival under stress conditions such as starvation, hypoxia, high temperatures, differentiation, protein metabolism, etc^2^.

The complex life cycle of human malaria parasite *P. falciparum* consists of three major stages; in mosquito gut, human liver and blood. During its 48 h asexual intraerythrocytic cycle, the parasite development progresses through ring, trophozoite and schizont stages producing upto 32 daughter cells (merozoites) that can reinvade fresh red blood cells. Some of the released merozoites develop into male and female gametocytes, which when taken up by a female mosquito during a blood meal undergo sexual reproduction in the mosquito midgut. The sporozoites which develop in the mosquito are released during the next mosquito bite and reach human hepatocytes. The sporozoites undergo another asexual division in liver and release merozoites into the blood stream, thus completing the cycle. Thus, throughout the life cycle, the parasite encounters phases of nutritional limitations and other stresses. Understanding pathways such as autophagy in this important parasite therefore may give insights towards developing novel antimalarials.

Using bioinformatics analyses, a limited number of autophagy proteins in *P. falciparum* genome have been identified^3,4^, but the precise functions of these putative proteins and the role of autophagy in this organism till date remains unanswered. Atg8, the autophagosome marker is the major autophagy protein that has been studied both in *P. berghei* as well as in *P. falciparum* in certain detail. Studies that investigated localization of this protein in *P. falciparum* have not yielded a clear picture. *Pf*Atg8 has been shown to localize exclusively or partially to the apicoplast^5,6^, in the host RBCs^6^ and also to be present as puncta in the parasite cytosol^4,6–8^. The localization of *Pf*Atg8 is shown to be unperturbed upon pharmacological disruption of PtdIns3K by wortmannin^5^, CQ^4,6^, inhibition of putative TOR by rapamycin^6^ or manipulation of the food vacuole pH^4^. Further, the absence of autophagy induction upon starvation is attributed to the lack of the negative regulator, TOR in *Plasmodium*^4,6^, although, Langsley *et al*. report^8^ increase in colocalization of *Pf*Atg8 and *Pf*Rab7 upon starvation.

Studies in *P. berghei* have indicated that in the liver stages, autophagy-like mechanism may be prevalent during metamorphosis of the sporozoites into merozoites to remove unnecessary organelles^9^. While in *P. falciparum*, it has been proposed that autophagy has evolved to perform multiple functions such as apicoplast biogenesis, protein turnover, cellular differentiation and partitioning into daughter cells^6^, but no experimental evidence exists for this hypothesis. The role of autophagy pathway during intraerythrocytic cycle thus has not been unequivocally established in *P. falciparum*. Furthermore, the localization and expression of *Pf*Atg8 is controversial and other autophagosome markers such as *Pf*Atg5 have not been explored yet. For these reasons, we initiated our studies to decipher the functional role of autophagy in *P. falciparum* during its erythrocytic stages and to characterize the putative *Pf*Atg5 as a player of *Plasmodium* autophagy pathway. We show that pharmacological inhibition of basal autophagy leads to compromised development and reinvasion abilities of the parasite. In addition, we also demonstrate that starvation-induced autophagy is temporally governed. Moderate starvation enables the parasite survival but prolonged starvation results in cell death. At the molecular level, both *Pf*Atg8 and *Pf*Atg5 appear as punctae resembling autophagosomes and their expression increase transiently upon brief starvation but later decrease with time which correlates with parasite survival and death respectively. Our study thus identifies a context dependent role of starvation-induced autophagy in this parasite.

## Results

### Inhibition of basal autophagy results in reduced reinvasion and *Pf*Atg8 expression

**Fig. 1:**
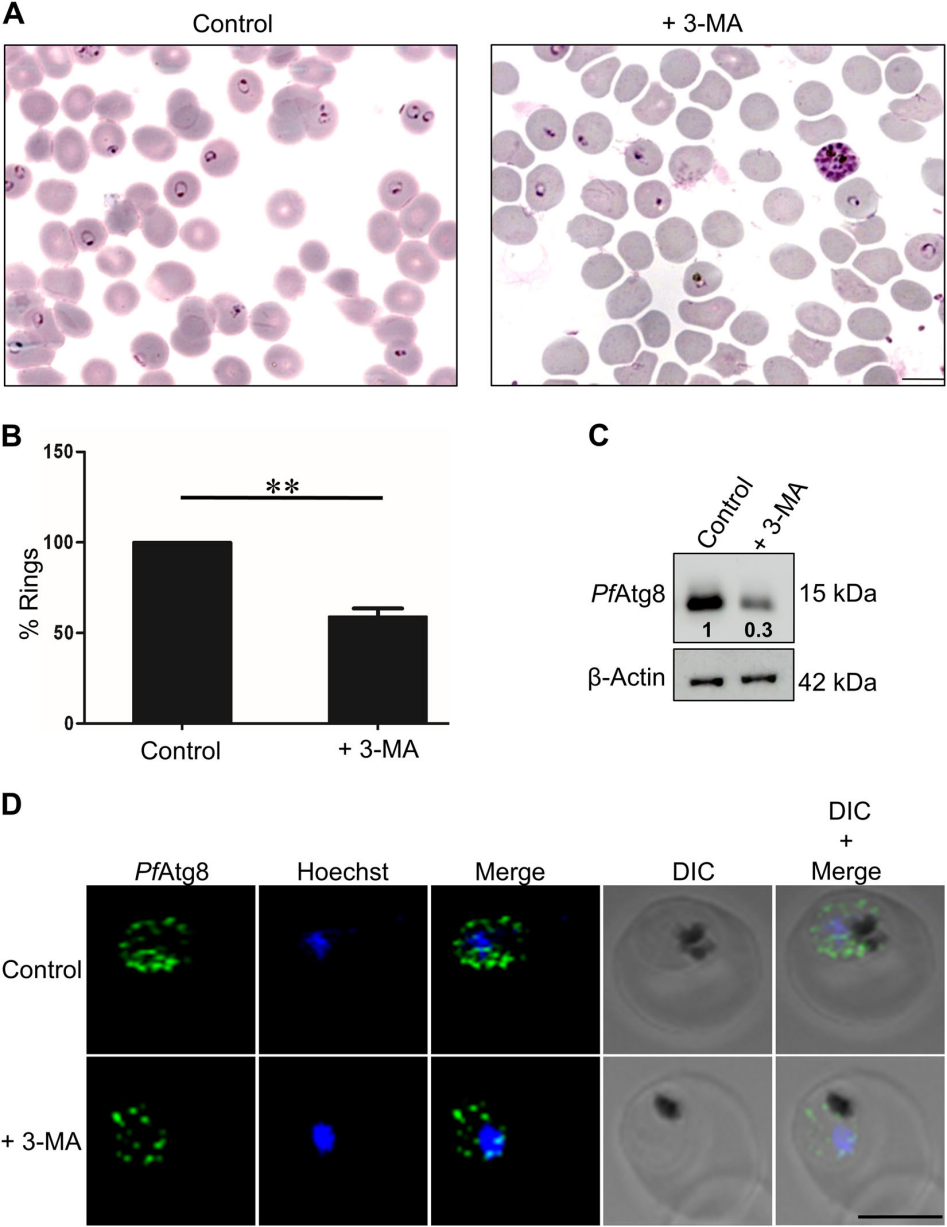
Inhibition of basal autophagy results in reduced reinvasion and *Pf*Atg8 expression. **a** Giemsa stained smears showing rings in control and 3-MA treated parasites. Highly synchronized parasites at late schizonts stage (38 h.p.i) were incubated in complete medium (control) with or without 3-MA (5 mM) for 2 h, and invasion was monitored by counting number of rings in the next cycle. Scale bar: 10 µm. **b** Graph representing percent rings in control and 3-MA treated parasites. The data presented are mean of 5 individual experiments. Error bars show the standard deviation. **P *<* 0.05. **c** Immunoblot analysis of *Pf*Atg8 expression levels. Lysates were prepared from tightly synchronized parasites at trophozoite stage incubated in complete medium with or without 3-MA for 2 h. Immunoblot was probed using custom generated anti-*Pf*Atg8 antibodies.β-actin was probed as loading control. Number below the bands indicate fold difference as compared to the normalized control. Uncropped blots are shown in Supplementary Figure 5. **d** *Pf*Atg8 immunofluorescence staining in control and 3-MA treated parasites. Hoechst was used as a DNA marker. Scale bar: 5 µm

A basal level of autophagy is responsible for maintaining cellular homeostasis in almost all the living organisms^1^. Despite a number of studies carried out recently, the role of autophagy in *P. falciparum* is poorly understood. To investigate the precise role of autophagy in *P. falciparum*, we tested the ability of blood stage parasites to invade fresh RBCs as an indicator of growth and development in presence or absence of the known autophagy inhibitor, 3-methyl adenine (3-MA). Synchronized schizont stage parasites when incubated with 3-MA showed 40% less rings compared to control in the ensuing invasion (Fig. 1a, b). Further, the expression levels of the autophagosome marker, *Pf*Atg8 in 3-MA treated parasites (trophozoite stage) were reduced by 3-fold (Fig. 1c). Immunocytochemical analysis using *Pf*Atg8 antibodies revealed the presence of Atg8 labeled vesicles (puncta)only in the parasite cytosol. The number of these Atg8 vesicles were reduced 50% in the 3-MA treated parasites as compared to control (Fig. 1d) corroborating our immunoblot analysis. To visualize these puncta more clearly, we used super resolution microscopy (Three-Dimensional Structured Illumination Microscopy, 3D-SIM). Numerous small vesicles labeled with *Pf*Atg8, resembling autophagosomes, were seen in the parasite cytosol in all the three stages, that is, ring, trophozoite, and schizont (Supplementary Figure S1). In addition, electron micrographs of starved parasites revealed the presence of *Pf*Atg8 on double/multiple membrane vesicles reminiscent of autophagosomes near and within the food vacuole (Supplementary Figure S2). As these vesicles have been shown to associate with the apicoplast, we studied the colocalization of *Pf*Atg8 vesicles with the apicoplast marker, Single-Stranded DNA Binding protein (SSB). These markers showed marginal colocalization (Supplementary Video S1 A and B). As 3-MA inhibits PI3Kinase which is upstream of the autophagy pathway and is also implicated in other functions, we studied the effect of MRT 68921, the only known specific autophagy inhibitor ULK1^10^. Activation of ULK1 initiates autophagy. *Pf*Atg1 gene ID *Pf*3D7_1450000 has been predicted to be a *Plasmodium* homolog of human ULK1^11^. We modeled the structure of *Pf*Atg1 with bound MRT 68921 (Supplementary Figure 3A,B). The docked inhibitor fits well into the binding pocket with an interaction energy of -7.8 kcal/mol. Superposition of the *Pf*Atg1 model along with the docked MRT68921 on the crystal structure of Human ULK1 kinase in complex with an inhibitor (PDB code: 4WNO) shows that MRT68921 fits well into the binding site (Supplementary Figure 3A). The binding site residues Lys70, Asp190, Phe191 are conserved between the two proteins and the gate keeper residue methionine is replaced by Leu123 in *Pf*Atg1. In the ULK1 kinase, a few hydrogen bonds are formed between the protein and the ligand. However, similar hydrogen bonds cannot occur with MRT68921 as the chemical structures of the two inhibitors are different. MRT68921 is likely to make different set of hydrogen bonds or water bridges with *Pf*Atg1, and the additional long chain will enhance binding by making more van der Waals interactions with the protein. We also examined the effect of this inhibitor on the parasite invasion. Our results show that MRT86921 inhibitor (200 nM) completely inhibits the invasion of the fresh erythrocytes (Supplementary Figure 3B). Altogether, these results verify our proposition that basal autophagy mediates the invasion in *P. falciparum*.

### Starvation-induced autophagy mediates parasite invasion

**Fig. 2:**
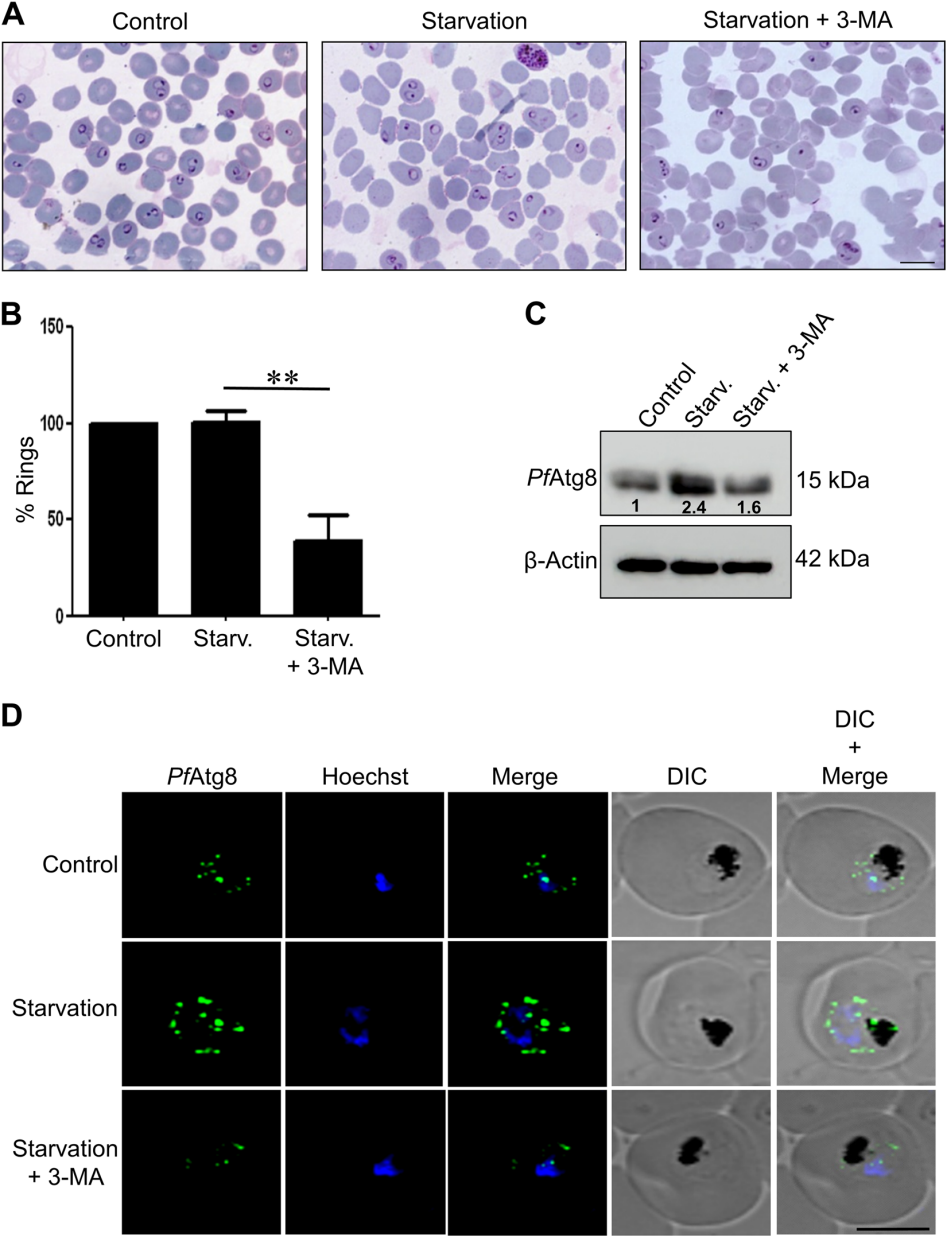
Starvation-induced autophagy mediates parasite invasion. **a** Giemsa stained smears showing rings in ensuing invasion of fresh RBCs in control, starved and starved parasites treated with 3-MA (5 mM, 2 h). Highly synchronized parasites at late schizont stage (38 h.p.i) were incubated in control, starvation medium (complete medium devoid of serum and amino acids) with or without 3-MA (5 mM) for 2 h, and number of rings in parasites were counted in ensuing invasion. Scale bar:10 µm. **b** Graph representing percent rings in parasites grown in control, starvation or starvation medium with 3-MA. The data represented are mean of 5 individual experiments (200 parasites counted in each experiment). Error bars show standard deviation. **P *<* 0.05. **c** Immunoblot analysis of *Pf*Atg8 expression levels. Lysates from trophozoites were prepared from control or starved parasites treated with or without 3-MA and probed using anti-*Pf*Atg8 antibodies.β-actin was used as loading control. Number below the bands indicate fold difference compared to the normalized control. Uncropped blots are shown in Supplementary Figure 5. **d** Immunofluorescence staining of *Pf*Atg8 in parasites incubated for 2 h in control, starvation or starvation medium with 3-MA (5 mM). Hoechst was used as a DNA marker. Scale bar: 5 µm

Nutrient deprivation invariably induces autophagy^12^. However, absence of autophagy induction by starvation has been reported in *P. falciparum* by several groups^4,6^. Since in our studies 3-MA-treated parasites showed reduced invasion and decreased *Pf*Atg8 expression, indicating that parasite autophagy can be modulated, we next addressed whether the parasite responds to nutrient limitations and if autophagy can be induced by starvation. Interestingly, late schizonts (38 h.p.i) when incubated in starvation medium for 2 h did not lead to a decrease in invasion competence as envisaged by the number of rings in the ensuing cycle (Fig. 2a, b). However, when the parasites were grown in starvation media along with 3-MA, 40% less rings were observed. The modulation of autophagy by starvation or 3-MA was also corroborated by monitoring *Pf*Atg8 expression by immunoblotting and immunofluorescence *Pf*Atg8 expression increased 2-fold upon starvation, while this rise was reduced to near basal levels in presence of 3-MA (Fig. 2c, d). These results demonstrate that autophagy in *P. falciparum* is induced in response to nutrient limitations mediating reinvasion of host RBCs.

### Starvation-induced autophagy is context dependent and determines parasite survival

**Fig. 3:**
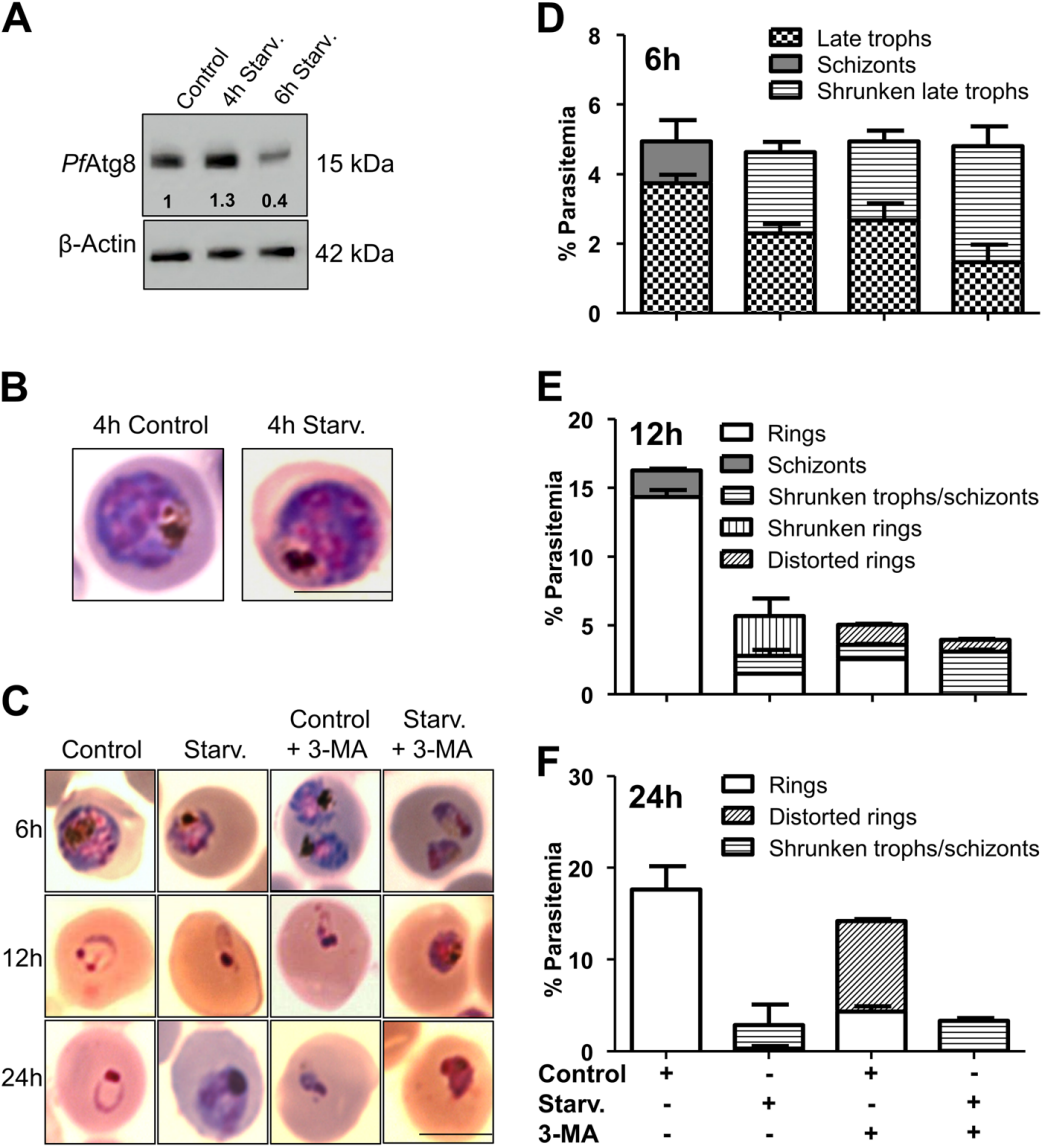
Duration of starvation-induced autophagy determines parasite survival. **a** Immunoblot analysis of *Pf*Atg8 expression levels upon 4 and 6 h starvation. Lysates from trophozoite stage parasites cultured in complete or starvation medium for 4 and 6 h were analyzed using immunoblotting by probing with custom generated anti-*Pf*Atg8 antibodies. Uncropped blots are shown in Supplementary Figure 6. **b** Morphological features of control and 4 h starved parasites stained with Giemsa. Scale bar: 5 µm. **c** Prolonged starvation induces abnormal morphology. Parasites at late trophozoite stage were incubated in control, control + 3-MA, starvation or starvation + 3-MA media for 6, 12 and 24 h. Parasite morphology was assessed in Giemsa stained smears. Scale bar:5 µm. **d**–**f** Graph representing parasites with starvation-induced morphological changes after 6, 12 and 24 h incubation in control, control + 3-MA, starvation or starvation + 3-MA media. Data represented is mean of 5 individual experiments. Number of parasites scored for morphology, *n* = 2500. Error bars show standard deviation

As short-term starvation-induced autophagy sustained parasite development and invasion, we further probed if prolonged starvation too could support the erythrocytic cycle. We first determined the autophagy status by monitoring the *Pf*Atg8 expression levels at the trophozoite stage. As seen for 2 h of starvation, *Pf*Atg8 expression remained high till 4 h (Fig. 3a). The parasites also appeared morphologically healthy till 4 h of starvation (Fig. 3b). Interestingly, parasites when starved for longer periods (6 h), showed decreased *Pf*Atg8 expression as well as retarded growth [Fig. 3a, c 6 h, Control and Starvation (Starv.), and Supplementary Figure S3], indicating that starvation-induced autophagy is temporally governed and is context dependent. Thus, while brief starvation (2–4 h) induces autophagy and maintains parasite development, prolonged starvation (6 h and beyond) leads to retarded growth. These results led us to further assess the effect of long-term starvation and autophagy decrease (perhaps due to exhaustion of the autophagic process) on parasite development and invasion. The effect of starvation was apparent at 6 h itself. When starvation or autophagy inhibition was extended upto 6 h, reduced parasitemia, appearance of abnormal parasite morphology and decrease in *Pf*Atg8 expression was apparent. While morphology of control parasites appeared as evenly stained, well rounded, large, full trophozoites with clear and concentrated hemozoin as well as healthy schizonts (Control, 6 h in Fig. 3c, Supplementary Figure S3 and Control, 6 h in Fig. 3d), 6 h starved parasites were smaller, with uneven staining (Starvation (Starv.), 6 h in Fig. 3c and Supplementary Figure S3). At this time point, the parasitemia remained same even when basal or starvation-induced autophagy was decreased (Fig. 3d). However, the proportion of healthy parasites decreased considerably in the starved and 3-MA treated population. These treated parasites appeared as shrunken late trophozoites with uneven staining (Starv., Control + 3-MA, Starv. + 3-MA, 6 h, Fig. 3c, d and Supplementary Figure S3). Beyond 6 h, starvation and 3-MA treatment progressively increased the population of unhealthy parasites. After 12 h starvation, while control parasites developed into late schizonts and rings, 80% of the total starved parasites appeared as shrunken rings or trophozoites. The parasitemia was 58% less in starved parasites compared to the control (Control, Starv., 12 h, Fig. 3c, Supplementary Figure S3 and 3e). Parasites incubated in starvation medium + 3-MA for 12 h remained as vacuolated trophozoites and there was 73% drop in total parasitemia compared to control, while parasites incubated in complete medium + 3-MA showed 69% decrease in total parasitemia (Control + 3-MA, Starv. + 3-MA, 12 h, Fig. 3c, Supplementary Figure S3 and 3e). After 24 h starvation, all the parasites grown in control medium progressed to ring stage while the parasites grown in starvation medium showed abnormal morphology with uneven staining. Parasites incubated in starvation medium + 3-MA, were darkly stained and condensed, while parasites incubated in complete medium + 3-MA appeared unevenly stained with jagged borders (Control, Starv., Starv. + 3-MA, Control + 3-MA, 24 h, Fig. 3c, Supplementary Figure S3). As expected, the parasitemia in untreated parasites increased to 18%. In contrast, there was a massive reduction in parasitemia in starved parasites (80%), starved + 3-MA (82%), and in unstarved parasites + 3-MA (30%) (Fig. 3f).

Altogether, it is evident from these results that, upon long-term starvation, the parasites display abnormal morphology and stalled growth, supporting our notion that extended starvation results in down regulation of autophagy leading to cell death. As prolonged starvation and inhibition of autophagy resulted in an increase in the number of unhealthy parasites and a concomitant decrease in the appearance of healthy rings at the end of one developmental cycle, we wondered what the fate of these unhealthy parasites upon replenishment with fresh growth medium would be. For this purpose, treated or untreated parasites after 6, 12 and 24 h of starvation were replenished with complete growth medium and the growth of these parasites were followed till the control parasites developed into subsequent stage. Progressive starvation and/or autophagy inhibition that resulted in stunted development and accumulation of unhealthy parasites were revived only to a certain extent compared to control, despite providing favorable nutrient-rich conditions (Supplementary Figure S4).

### Long-term starvation inhibits reinvasion of host RBCs

**Fig. 4:**
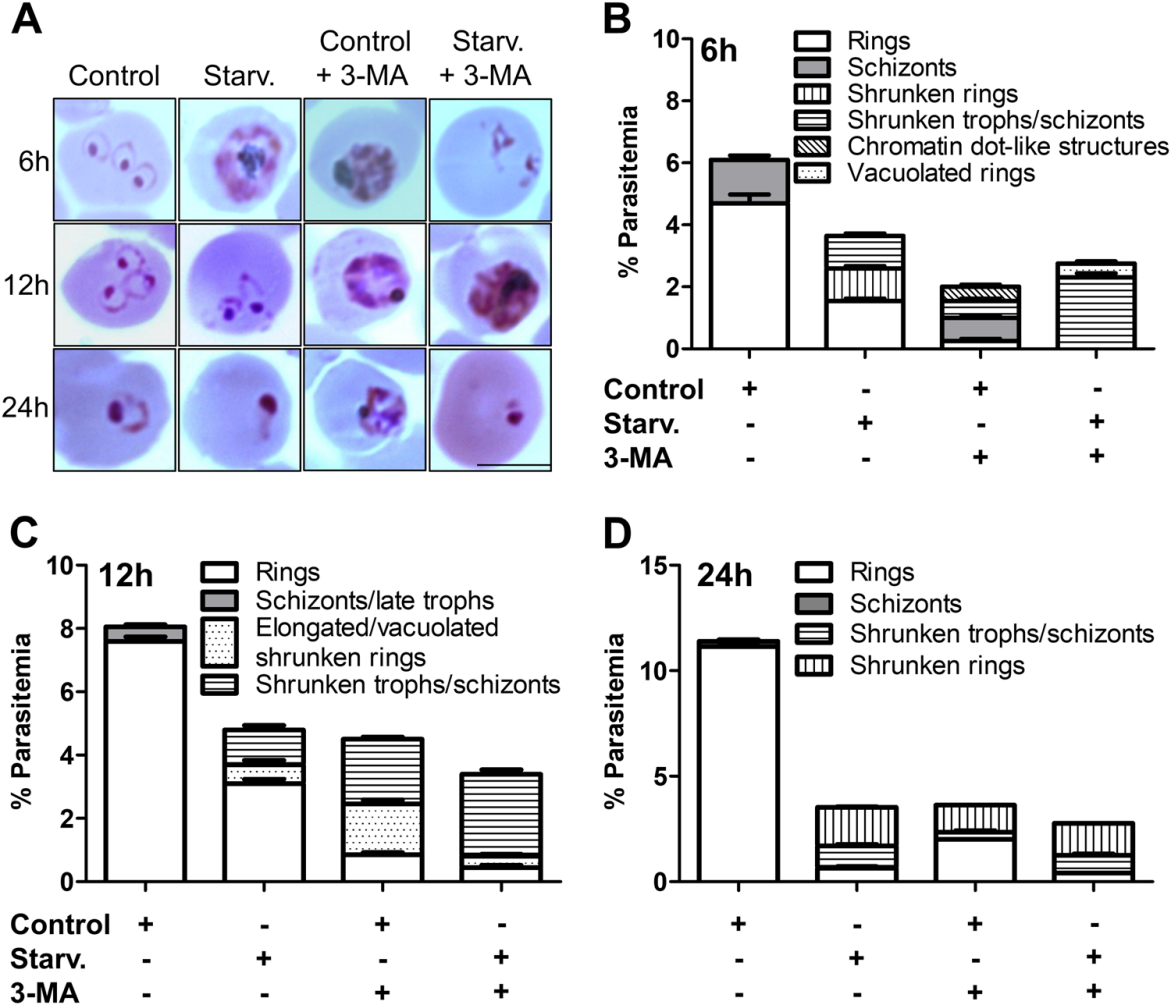
Long-term starvation inhibits reinvasion of host RBCs. **a** Giemsa stained smears showing rings in representative iRBCs. Highly synchronized schizonts were incubated in complete or starvation medium in presence or absence of 3-MA for 6, 12 and 24 h. Giemsa stained smears were assessed for number of rings and parasite morphology. Scale bar: 5 µm. **b** As described in (**a**), morphological analysis of the parasites after various treatments at different time points were carried out. Graphs representing % parasitemia for these three time points are shown in **b**–**d**. The data represented are mean of 5 individual experiments. Number of parasites scored for rings, *n* = 2500. Error bars show standard deviation

We next determined the effect of long-term starvation on invasion by following the development of late trophozoites/schizonts to rings in the next cycle. This progression was monitored by incubating late trophozoites/schizonts in complete (control) or starvation media, both in presence and absence of the autophagy inhibitor 3-MA for 6, 12 and 24 h. While most of the control parasites developed into rings post 6 h, many starved parasites remain arrested as late trophozoites/schizonts (Control and Starv., 6 h, Fig. 4a). Quantitation of parasitemia after 6 h revealed a 40% decrease in starved parasites as compared to control (Control and Starv., 6 h, Fig. 4b). Morphologically, while in control, 80% of the total parasites progressed to rings, only 40% healthy rings were observed with rest appearing as shrunken parasites under starved conditions (Control and Starv., 6 h, Fig. 4a, b). In parasites incubated in complete medium with 3-MA for 6 h, there was a 63% decrease in parasitemia as compared to control, while presence of 3-MA in starvation medium reduced the parasitemia by 28% as compared to starvation medium alone (Control + 3-MA and Starv. + 3-MA, 6 h, Fig. 4b). Complete medium with 3-MA showed along with a few healthy rings, abnormal parasite morphology such as rings with multiple chromatin-like dots (17%)and condensed schizonts with dark staining (26%). In parasites incubated in starvation medium + 3-MA, none of the parasites appeared morphologically normal; 82% parasites were condensed schizonts and the rest were rings with chromatin-like dots in this group (Fig. 4a). The effect of 12 and 24 h starvation was much more deleterious on the invasion efficiency. As compared with control, there was a 37% drop in parasitemia at 12 h which after 24 h further decreased to 67% (Fig. 4c, d). Extended starvation also resulted in a temporal increase of unhealthy parasites. After 12 h, 95% of the parasites grown in complete medium were healthy rings which were only 63% in starved parasites (Fig. 4a). Shrunken trophozoites/schizonts and elongated/vacuolated rings constituted the rest of the starved parasites. Inhibition of autophagy by 3-MA during prolonged starvation (12 and 24 h) further exacerbated the morphological abnormalities of the parasites resulting in reduced invasion fitness. Treatment with 3-MA in control medium caused a 42% decrease in parasitemia as compared to control at 12 h, which further slid to 67% at 24 h. Similarly, although there were barely unhealthy rings at 12 and 24 h in control, their numbers were 36% in 12 and 24 h in presence of 3-MA in complete medium. The unhealthy rings exhibited various deformities appearing as either shrunken, elongated with flattened nuclei, vacuolated or seen as chromatin-like dots without any ring structure. Remaining parasites in this group were late trophozoites with dense and condensed cytoplasm. There was a 58 and 74% drop in total parasitemia in parasites grown in starvation medium with 3-MA for 12 and 24 h respectively, as compared to control (Fig. 4c, d). Of the parasites that were cultured for 12 and 24 h in starvation medium in the presence of 3-MA, only 12–14% rings appeared normal. Rest of the abnormal parasites showed varied morphologies such as condensed schizonts with dark staining, or shrunken and vacuolated rings (Fig. 4a).

From these results, it is evident that prolonged starvation progressively results in reduced invasion. Blocking either basal or starvation-induced autophagy for an extended duration has a more telling effect on invasion as compared to starvation alone, further strengthening our notion that autophagy supports *P. falciparum* survival.

### Similar to *Pf*Atg8, the putative *Pf*Atg5 is expressed throughout the intraerythrocytic cycle as vesicles in the parasite cytosol

**Fig. 5:**
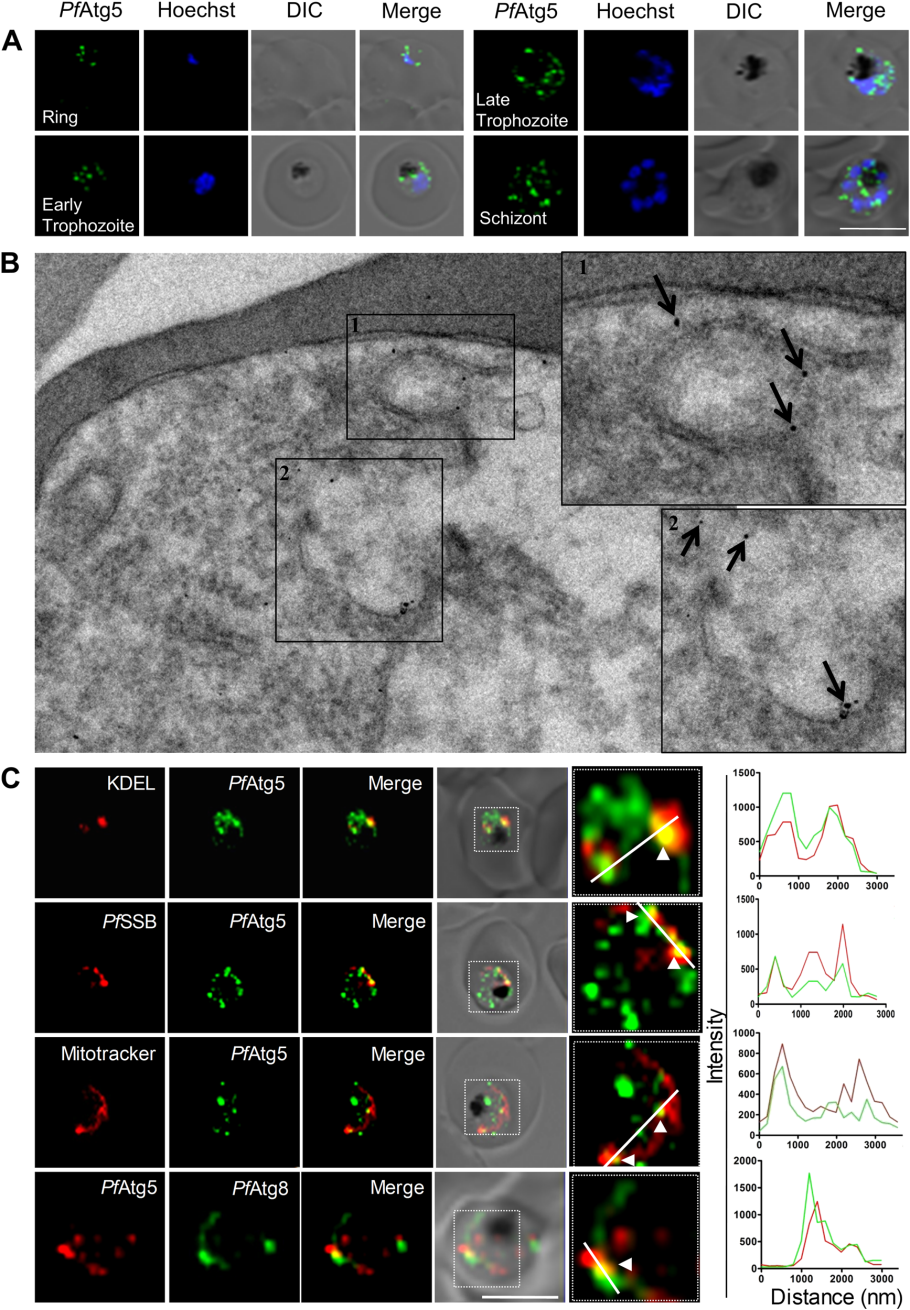
The putative *Pf*Atg5 is expressed throughout the intraerythrocytic cycle and appears as puncta in the parasite cytosol. **a** *Pf*Atg5 immunofluorescence staining at various intraerythrocytic stages. Tightly synchronized parasites at ring, early trophozoite, late trophozoite and schizont stage were immunolabeled with anti-*Pf*Atg5 antibodies. Representative image from at least 20 parasites is shown. Hoechst was used as a DNA marker. Scale bar: 5 µm. **b** RBCs infected with *P. falciparum* (8% parasitemia) were analyzed by immunoelectron microscopy (immunogold and silver enhancement method) with antibody against *Pf*Atg5. Insets 1 and 2 show enlarged images of Atg5 decorated double membrane structures. Scale bar: 0.2 μm. **c** Synchronized parasites at trophozoite stage were immunolabeled with anti-*Pf*Atg5 antibodies (1:400) along with various organelle markers: Anti-KDEL antibody (1:200) as an endoplasmic reticulum marker, Anti-*Pf*SSB antibody (1:200) as an apicoplast marker, MitoTracker Red CMXROS (100 nM) as a marker for mitochondria and Anti-*Pf*Atg8 antibody as a marker for autophagosomes. Hoechst was used as DNA marker; Scale bar: 5 µm

As perturbation of autophagy showed dramatic effects on development, invasion and parasite survival, we wanted to explore further molecular details of autophagy in terms of autophagosome formation in *P. falciparum*. Atg8 has been extensively used as a late stage autophagosome marker in all model organisms and also in *Plasmodium* spp. However, early-stage autophagy markers such as Atg5 and Atg12 have not been explored in *Plasmodium*, although these have been identified in its genome^5^. Using antibodies against the putative *Pf*Atg5, immunocytochemical staining revealed the presence of *Pf*Atg5 decorated vesicles in the parasite cytosol in ring, trophozoite and schizont stages (Fig. 5a). Super resolution microscopy (3D-SIM) too revealed the presence of such structures (Supplementary Figure S1B). Interestingly, these *Pf*Atg5 vesicles appeared similar to the *Pf*Atg8 vesicles resembling autophagosomes (Figs. 1d, 2d). In addition, the presence of these Atg5 labeled double membrane vesicles was confirmed by immunoelectron microscopy (Fig. 5b). In complex eukaryotes, most autophagosomes appear to be generated from or are very close to, the endoplasmic reticulum. However, mitochondria, plasma membrane and Golgi have also been reported to contribute membranes for autophagosome generation^13–15^. We therefore investigated the colocalization between the early autophagosome marker *Pf*Atg5 with various *Plasmodium* organelle markers such as KDEL (ER), SSB (apicoplast), MitoTracker Red (mitochondria) and also with the *Pf*Atg8 (mature autophagosomes). We observed that *Pf*Atg5 partially colocalized with ER, mitochondria, apicoplast and *Pf*Atg8 (Fig. 5c).

### *Pf*Atg5 expression too is induced by short-term starvation and inhibited by 3-MA

**Fig. 6:**
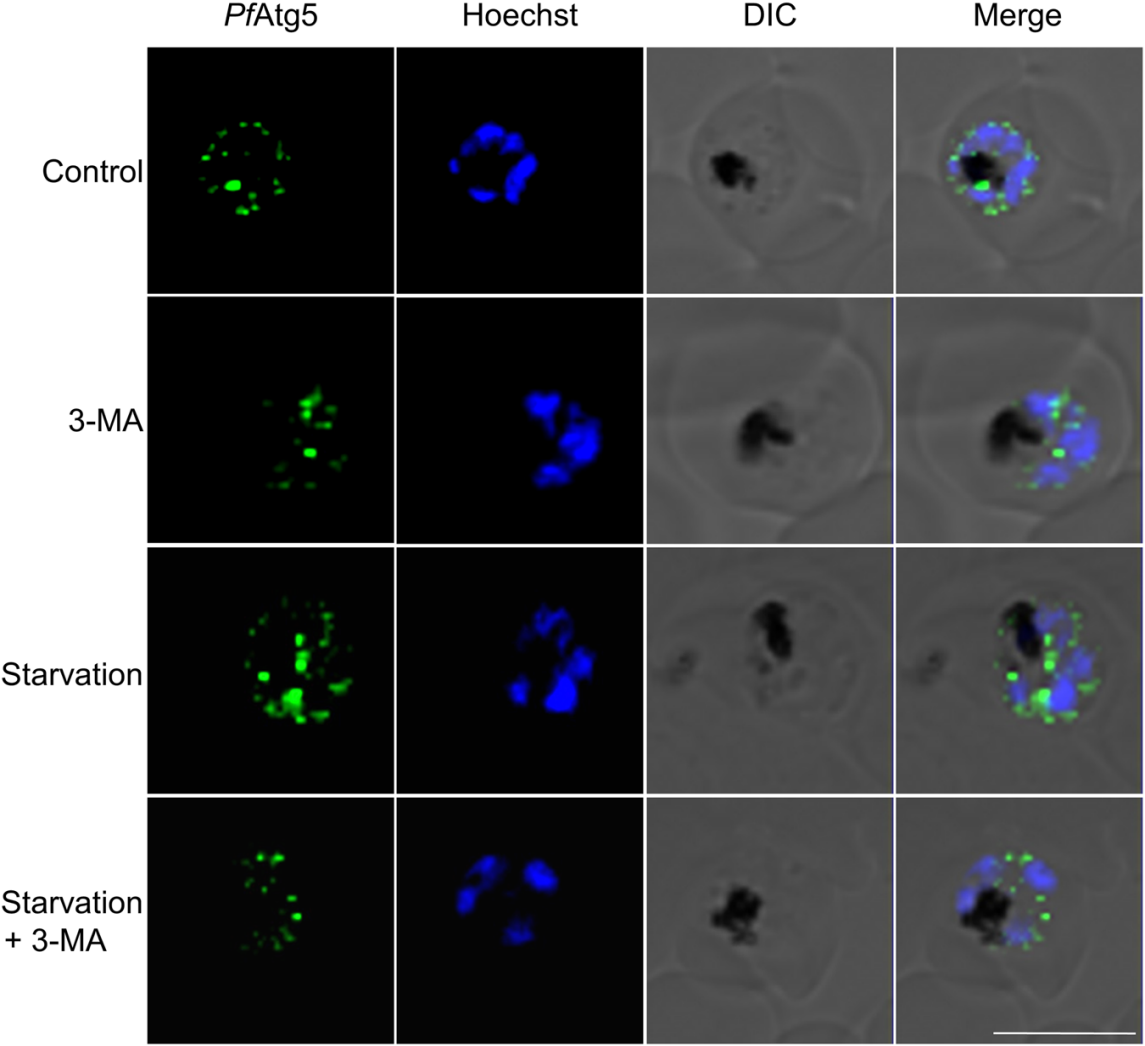
*Pf*Atg5 expression too is induced by short-term starvation and inhibited by 3-MA. Immunofluorescence staining of *Pf*Atg5 in parasites incubated in control, starvation, control + 3-MA or starvation + 3-MA media for 2 h. Representative image from at least 20 parasites is shown. Hoechst was used as DNA marker. Scale bar: 5 µm

Since, we observed that transient starvation-induced *Pf*Atg8 expression while blocking autophagy reduced it, we wanted to see if *Pf*Atg5 expression pattern mimics that of *Pf*Atg8. As seen for *Pf*Atg8, we observed that *Pf*Atg5 expression too increased upon starvation for brief periods (2 h, Fig. 6). This result was reflected in the appearance of *Pf*Atg5 vesicles in late trophozoites/schizonts that were subjected to starvation in presence or absence of autophagy inhibition by 3-MA. Starvation caused an increase in abundance of *Pf*Atg5 puncta while it diminished upon 3-MA treatment in normal growth conditions or during starvation (Fig. 6).

## Discussion

This study experimentally establishes the role of basal and starvation-induced autophagy in mediating *P. falciparum* development and invasion during its intraerythrocytic stages, demonstrating a prosurvival mechanism unlike reported for other apicomplexans such as *Trypanosoma brucei*^16^, and *Toxoplasma gondii*^17,18^. To address the role of autophagy in depth, we investigated the consequence of basal autophagy inhibition on blood stage parasite cycle over short-term and extended starvation. As earlier studies using genetic manipulation approaches indicated that *Plasmodium* Atg8^3^ and *Pf*Atg7^19^ are essential for the parasite survival, we therefore chose to use alternative strategy such as pharmacological tools (autophagy inhibitors and inducers) to investigate the role of *Plasmodium* autophagy.

First, we addressed whether *Plasmodium* autophagy gets induced upon starvation as there have been conflicting reports. Studies on absence of autophagy induction are supported by the observation that bioinformatic analyses have not identified the complete set of the core autophagy machinery genes including Atg13, Atg9 and Atg16^11^, among others in *P. falciparum*, and in addition, starved parasites too have not shown induction in *Pf*Atg8 levels^4^. However, a recent study showed increased colocalization of *Pf*Rab7and *Pf*Atg8 positive vesicles upon 4 h of amino acid starvation^8^. We demonstrate that the parasite indeed responds to nutrient limitation by upregulating autophagy albeit for short duration and that this starvation-induced autophagy is context specific. While mild starvation induces autophagy enabling parasite development and invasion, prolonged starvation leads to decreased expression of *Pf*Atg8, compromising autophagy which in turn leads to stalled growth and reduced invasion. Thus, our studies demonstrate induction of autophagy despite the lack of experimental evidence of a functional of TOR homolog in *P. falciparum*. Further, in the presumptive absence of Atg13, it would be interesting to explore that *Pf*Atg1 participates in autophagy independently or in concert with FIP200 and Atg101^11^.

Induction of autophagy is documented by monitoring the expression of the autophagosome marker *Pf*Atg8 protein levels and the number and intensity of the puncta seen upon starvation. Again, in the literature the distribution of these *Pf*Atg8 puncta is controversial. While some studies have shown them to localize exclusively to the apicoplast^5^, others have observed their presence within RBC cytosol^6^. We approached to resolve this issue by using heterologous (commercial LC3 A and B) as well as endogenous (*Pf*Atg8) antibodies in immunoblotting and immunofluorescence techniques across all stages of the parasite erythrocytic cycle during starvation and/or autophagy inhibition (data with heterologous antibodies not shown). To further substantiate our results, we also employed another hitherto unexplored autophagosome marker in *P. falciparum*, the putative *Pf*Atg5 and carried out similar analysis as mentioned for *Pf*Atg8. In all these experiments, we consistently made two observations: one, transient or short starvation periods led to an upregulation of *Pf*Atg8 and *Pf*Atg5 expression with a concomitant increase in autophagosome numbers which was repressed upon autophagy inhibition; two, the distribution of the puncta resembling autophagosomes under all conditions and stages was restricted to the parasite cytosol alone. Discrepancies in non-induction of autophagy by starvation as well as redistribution of *Pf*Atg8 vesicles reported in the past could be due to several reasons. Firstly, media used for such studies have varying compositions. While majority of the previous studies have used minimal medium^6^ or Hanks’ Balanced Salt Solution (HBSS)^4^, the starvation medium used by us is RPMI devoid of all amino acids and serum. Secondly, since maximum *Pf*Atg8 expression was observed at late trophozoite stage, all our studies were carried out at this stage, while young/mid trophozoites were mostly used by others^4,6^. Also, our results show that autophagy induction is sustained only upto 4 h of starvation and beyond this period *Pf*Atg8 levels are decreased leading to compromised parasite survival. In most of the studies carried out earlier, starvation was for 6 h (except in the studies of Tomlins et al^8^, where parasites were cultured in 1% O_2_, 3% CO_2_, 96% N_2_, and thus the results could be the combined effect of starvation and hypoxia).

Further, in absence of the full repertoire of core autophagy proteins as suggested by bioinformatics analyses, it has been difficult to envisage a functional autophagy pathway in this parasite. However, we find several lines of evidence in our studies that strongly point towards an operational and responsive autophagy pathway. First, we find that the parasite succumbs to starvation pressure when basal autophagy is inhibited. Second, brief starvation leads to transient increase in levels and puncta of both the autophagosomal markers, *Pf*Atg8 as well as *Pf*Atg5. Importantly, both *Pf*Atg8 and *Pf*Atg5 appear as puncta throughout the parasite cytosol, suggesting the presence of autophagosomes like vesicles that partially colocalizes with organelles such as mitochondria and ER that are known to supply membranes during autophagosome biogenesis^15^. Third, parasite cycle is sustained during short-term starvation and this cycle collapses upon autophagy inhibition. Fourth, prolonged starvation reduced the expression of the autophagy markers with concomitant parasite death.

Our characterization of the putative *Pf*Atg5 unravels several unexpected and unique observations. As noted for mammalian Atg5^14^, we show that *Pf*Atg5 too appears as puncta in the cytoplasm of the parasite that partially colocalizes with *Pf*Atg8, ER and mitochondria. Taken together, these results suggest that *Pf*Atg5 also participates in the autophagy pathway. Future work will be aimed at detailed studies in determining if a functional *Pf*Atg5–*Pf*Atg12 complex exists. At the molecular level, there are a few deviations from the classical Atg5-Atg12 seen in other species. The putative *Pf*Atg5 is much longer protein as compared to its counterparts and although the lysine involved in conjugation with *Pf*Atg12 is present at position 479, in addition, a lysine residue is present at position 480^5^. Whether conjugation of *Pf*Atg5 with the putative *Pf*Atg12 in absence of the glycine at the C terminus of the *Pf*Atg12 occurs or it conjugates with the inner glycine at 110 position of *Pf*Atg12 needs further investigation. Interestingly, homolog of Atg16 has not yet been identified. Further work will shed light if this conjugation system and a functional canonical complex exists or whether *Pf*Atg5 tethers to the membrane vesicles independent of *Pf*Atg12 as has been suggested for the yeast homolog^20^. Finally, in absence of a functional conjugation system, the possibility of these proteins engaging in other trafficking pathways cannot be ruled out.

Perhaps the more pertinent question to be asked is the need for autophagy in this parasite. The malaria parasite undergoes three major development stages during its intraerythrocytic cycle needing large and consistent supply of nutrients. The requirement of nutrients by the parasite during this asexual phase of life is reflected by the fact that almost 70–80% of hemoglobin is degraded by the parasite during the trophozoite stage to meet its demand for amino acids^21^. Also, in physiological context, during *P. falciparum* infection, the parasites get sequestered in blood vessels of various organs and as a result, the blood supply is obstructed and parasites do face severe nutrient limitation. Considering these scenarios and our results, it appears that autophagy serves as prosurvival mechanism under such conditions.

Altogether, our studies provide insights into the vital role of basal and starvation-induced autophagy in *P. falciparum* growth, development and invasion. For this, the parasite has evolved a unique autophagy machinery with a limited set of proteins with characteristic features. It is also tempting to suggest that these putative autophagy proteins may also have non-autophagy roles such as those ascribed for Atg8 in apicoplast biogenesis^5^. A comprehensive exploration to assign mechanistic roles of various proteins involved at different steps of autophagy in the parasite will need further exploration for developing novel therapeutics.

## Materials and Methods

### *P. falciparum* parasite culture

In vitro parasite culture procedures were approved by the Institutional Human Bioethics and Biosafety Review Committee of Jawaharlal Nehru Centre for Advanced Scientific Research, Bangalore, India. *Plasmodium falciparum* 3D7 strain was cultured in human O^+^ erythrocytes at 37 °C under 5% O_2,_ 5% CO_2_ and 90% N_2_ in RPMI 1640 (Sigma) supplemented with 27 mM sodium bicarbonate, 11 mM glucose, 0.37 mM hypoxanthine, 10 μg/ml gentamicin and 10% heat inactivated human serum as described previously^22^. Parasite cultures were synchronized by sorbitol treatment^23^.

### Invasion assay

Synchronized parasites at late schizont stage (38 h.p.i) were cultured in complete or starvation medium (RPMI 1640 without amino acids and serum, HyClone Laboratories Inc., South Logan, Utah) with or without 5 mM 3-MA (M9281, Sigma). Invasion in the next cycle was checked by counting the number of rings in Giemsa stained thin smears using Axiostar plus microscope (Carl Zeiss).

### Immunoblot analysis

For immunoblotting, synchronized parasites were separated from erythrocytes by treatment with 0.15% saponin in Phosphate Buffered Saline (PBS) for 15 min at 37 °C followed by washes in cold PBS and centrifugation at 10,000 × *g* for 10 min. Saponin- treated parasites were suspended in SDS-PAGE sample buffer for 30 min at room temperature followed by sonication at 30 watts, 2 sec pulses three times at 5 min interval. Lysates were centrifuged at 16,000 × *g* for 5 min and supernatant was collected. Protein was estimated by Bradford method^24^. Equal amount of protein (30 μg) was resolved by 6 M urea-SDS-PAGE gel for *Pf*Atg8 and 10% SDS-PAGE for *Pf*Atg5 and blotted to PVDF membrane (Millipore). Primary antibodies used for immunodetection were custom generated rabbit anti-*Pf*Atg8 (1:1,000) raised against a peptide IPVVCERANRSNLPHEKKK (corresponding to amino acids28-47) anti-*Pf*Atg5 (1:1000) raised against a peptide corresponding to amino acids 170–190 (Genemed synthesis, US) and anti-β-actin (1:3000, Sigma) diluted in 5% skimmed milk/PBS. Both the custom antibodies were affinity purified. Their specificities were confirmed by peptide competition and by using uninfected RBC lysate. The commercial LC3 antibodies were LC3A (L8918, Sigma and 4599, Cell Signaling Technologies) and LC3B (3868, Cell Signaling Technologies). The signals were developed with an HRP-conjugated anti-rabbit secondary antibodies (1:3,000, Bangalore Genei) and Clarity Western ECL Substrate (Biorad) using Versa Doc (Biorad). Images were processed for brightness and contrast by Photoshop (Adobe Systems Inc.).

### Immunofluorescence Assay (IFA)

Immunofluorescence assays were performed as described earlier^25^. Parasites were washed twice with PBS and fixed using 4% paraformaldehyde (ProSciTech, Electron Microscopy-EM grade) and 0.0075% glutaraldehyde (EM grade) in PBS at room temperature for 30 min. Fixed cells were washed 2 times with PBS and permeabilized using 0.1% Triton X-100 at room temperature for 3 min. Cells were washed again twice with PBS followed by blocking with 3%BSA at 4 °C for 1 h. The primary antibodies were used at the following dilutions; rabbit anti-*Pf*Atg8 (1:600), rabbit anti-*Pf*Atg5 (1:400), mouse anti-SSB protein (1:400) and mouse anti-KDEL (1:400) in 3% BSA in PBS for 1 h at room temperature. Secondary antibodies used were Alexa Fluor 488- and 568-conjugated goat anti-rabbit and goat anti-mouse (Molecular Probes) respectively at 1:200 dilutions in 3% BSA for 1 h at room temperature. Nucleus was stained with Hoechst 33258 (Sigma). Parasites were mounted over the glass slide using ProLong Gold antifade mountant (Molecular Probes). Images were acquired with Carl Zeiss LSM700 or DeltaVision (Applied Precision, GE) microscope. Software used were Zen and SoftWoRx respectively using a 100X oil immersion phase contrast objective. DIC images were taken with polarized light.

### MitoTracker staining

Parasites were incubated with 100 nM MitoTracker Red CMXROS (M7512, Life Technologies) for 15 min at 37 °C, followed by washing the cells with PBS and fixing as described for IFA.

### *Pf*Atg8-Atg5 colocalization studies by Zenon complex

The parasites were fixed using 4% paraformaldehyde (ProSciTech, EM grade) and 0.0075% glutaraldehyde in PBS at room temperature for 30 min. Fixed cells were washed twice with PBS and permeabilized with 0.1% Triton X-100 in PBS for 5 min at room temperature. Following PBS washes, cells were blocked in PBS containing 3% BSA for 1 h at 4 °C. This protocol was followed for the first primary antibody anti-*Pf*Atg5 (1:200), Alexa Flour 568-conjugated goat anti-rabbit (1:200) was used as secondary antibody. Zenon rabbit IgG labeling was used to label *Pf*Atg8. Zenon complex IgG 488 was prepared as recommended by the supplier (Zenon® Alexa Fluor® 488 Rabbit IgG Labeling Kit, ThermoFisher Scientific) at a molar ratio of 6:1. Zenon complex thus formed was incubated for 1 h followed by fixation by 4% paraformaldehyde and 0.0075% glutaraldehyde in PBS at room temperature for 30 min. Nuclear staining was performed with Hoechst 33258 (1:300) for 10 min. Parasites were immediately mounted on the glass slide using VECTASHIELD (Vector Laboratories) mountant and imaging was carried out using Zeiss LSM 700 confocal microscope.

### Immunoelectron microscopy

For immunodetection of *Pf*Atg5 in infected RBCs, the previously described pre-embedding silver enhancement immunogold method^26^ was used with slight modifications.

The parasitized erythrocytes were fixed in 2% paraformaldehyde and 0.05% glutaraldehyde dissolved in 0.1 M sodium phosphate buffer (PB) (pH 7.4) for 2 h and then washed three times with PB. Then the cells were then resuspended in 2% agar and pelleted again. The cell pellets were immersed in 30% sucrose (wt/vol) overnight at 4 degrees. Immunolabeling was performed on 10-micron thick cryostat sections after blocking with 0.1% gelatin (wt/vol)-1% BSA (wt/vol) in 0.02 M PBS for 30 min (blocking buffer 1) followed by another blocking in 1% NGS in PBS-gelatin-BSA buffer (blocking buffer 2).

Sections were incubated for 2 h at room temperature with 1:10 dilution of rabbit anti-*Pf*Atg5 in blocking buffer 2. After washing steps, sections were incubated with ultra-small gold particles (1:50 dilution, Electron Microscopy Sciences) for 4 h at room temperature, followed by washing and post fixation with 2% glutaraldehyde for 20 min. Silver enhancement (R-GENT SE-EM, Electron Microscopy Sciences) was performed en-bloc for 10 minutes followed by dehydration in graded series of ethanol. Finally, the sections were embedded in Epon 812 resin and allowed to polymerize overnight at 60 °C. Ultrathin sections (70 nm thick) were cut on RMC ultramicrotome, stained with 1% Uranyl acetate and imaged in Tecnai G2 20 twin (FEI) transmission electron microscope.

## Electronic supplementary material

Supplemental legends

Supplementary figures

Supplemental Video S1A

Supplemental Video S1B
