# Supplementary material for: Basal and starvation-induced autophagy mediates parasite survival during intraerythrocytic stages of Plasmodium falciparum
Source: Cell Death Discov. 2018 Oct 3;4:43. doi: 10.1038/s41420-018-0107-9 (PMC6170468; doi:10.1038/s41420-018-0107-9)
Supplement: Supplementary file 1 — Supplemental legends [file 41420_2018_107_MOESM1_ESM.docx]

**Basal and starvation induced autophagy mediates parasite survival during intraerythrocytic stages of *Plasmodium falciparum*.**

Shiny Joy^1^, Lavanya Thirunavukkarasu^1^, Palak Agrawal^1^, Archana Singh^2^, B.K. Chandrasekhar Sagar^3^, Ravi Manjithaya^1^* and Namita Surolia^1^*

**Supplemental material**

**Figure S1.** (**A**)Localization of *Pf*Atg8 in different parasite stages by super resolution microscopy (3D-SIM). The distribution of endogenous *Pf*Atg8 is revealed in wild type parasites using antiserum raised against *Pf*Atg8 peptide. Parasites fixed and stained with antibodies were analyzed with Zeiss super-resolution system ELYRA PS1 with laser lines 405nm (DAPI) and 488nm (Alexafluor 488). Plan-Apo 63X oil-immersion/140 was used and the samples were imaged using the software ZENBLACK and processing was done by the module for SR-SIM. Scale bar: 2 µm. (**B**) *Pf*Atg5 localization using Structured Illumination Microscopy (3D-SIM), **s**howing *Pf*Atg5 decorated vesicles in parasite cytosol. Parasites fixed and stained with antibodies were analyzed with Zeiss super-resolution system 880 with Airyscan. Scale bar: 1 µm.

**Figure S2.** Immunoelectron microscopy localization of *Pf*Atg8. (**A-G**) Immunogold (5nm gold particles) and silver enhanced staining of starved *P. falciparum* reveals localization of *Pf*Atg8 on double/multimembrane bound organelles. (**A**) Parasite within host RBC showing *Pf*Atg8 decorated organelle. (**B and C**) Magnified of the area indicated in (**A**). (**D**) Another image of the parasite with *Pf*Atg8 signals within the food vacuole which is characterized by presence of haemozoin. (**E and F**) Magnified images of the area shown in (**D**). (**G**) Another magnified image showing *Pf*Atg8 signals within a food vacuole as indicated by distribution of 10 nm gold particles (arrows). R-RBC, P-parasite. Arrows indicate *Pf*Atg8 signals, yellow arrowheads indicate hemozoin crystals typical of a food vacuole. Scale bars (**A**) 0.5 µm, (**B**)0.2 µm, (**C**) 0.1 µm, (**D**) 0.2 µm, (**E-F**) 0.1 µm, (**G**) 0.2 µm.

**Figure S3**. (**A**) Homology model was generated using Phyre2 server.^1^ The MRT68921 dihydrochloride inhibitor (green) was docked on homology model of *Pf*Atg1 using AutoDock Vina.^2^(**B**) Human ULK1 is superposed on *Pf*Atg1 (cyan). The conserved residues Lys70, Asp190, Phe191 are shown as sticks in the binding pocket and the gate keeper residue methionine is replaced by Leu123 in *Pf*Atg1.Giemsa stained smears showing rings in untreated control, (**C**) and MRT68921 treated parasites (**D**). Highly synchronized late trophozoite were incubated in complete medium (Control) with or without MRT68921 (200nM) for 24h and invasion was monitored by counting numbers of rings in the next cycle.

**Figure S4.** Replenishment of starved parasites with complete medium after long term starvation revives parasite development only partially. **(A)**Control and starved parasites with and without 3-MA were incubated for 6, 12 and 24h, and were then replenished with complete medium till the control parasites progressed to rings or next developmental stage. Morphology was assessed by Geimsa staining. Scale bar:5 µm. **(B, C and D)** Graphs representing percent parasitemia determined for parasites as in **(A)**. Data represented is mean of three individual experiments. n=100, number of parasites scored in each experiment. Error bars show standard deviation.

**Figure S5.** Uncropped images of immunoblots appearing in Figures **1C, 2C, 3A**. The dotted line box indicates the lanes that are represented in the respective main figures.

**Video S1.** Localization of *Pf*Atg8 and apicoplast specific Single-stranded DNA binding protein (SSB). The distribution of endogenous *Pf*Atg8 (Green) and *Pf*SSB (Red) is shown in wild type parasites (trophozoites). For **VideoS1A** and **S1B,** IFA imaging was carried out using a LeicaSP8 confocal microscope. The images were acquired at512 X 512 resolution with spectral HyD detectors, with 100X/1.4 NA oil immersion objective. Software used was LAS X with 3D constructive module for processing. Laser lines were 488nm (Alexafluor 488) and 561nm (Alexafluor 568).

References

1. Kelley, L. A., Mezulis, S., Yates, C. M., Wass, M. N. & Sternberg, M. J. E. The Phyre2 web portal for protein modeling, prediction and analysis. *Nat. Protoc.* **10,** 845-858 (2015).

2. Trott O. & Olson A. J. AutoDock Vina: Improving the speed and accuracy of docking with a new scoring function, efficient optimization, and multithreading. *J. Comput. Chem.* **31,** 455-461 (2009).
