# Supplementary material for: Basal and starvation-induced autophagy mediates parasite survival during intraerythrocytic stages of Plasmodium falciparum
Source: Cell Death Discov. 2018 Oct 3;4:43. doi: 10.1038/s41420-018-0107-9 (PMC6170468; doi:10.1038/s41420-018-0107-9)
Supplement: Supplementary file 2 — Supplementary figures [file 41420_2018_107_MOESM2_ESM.pdf]

Supplementary Figure S1

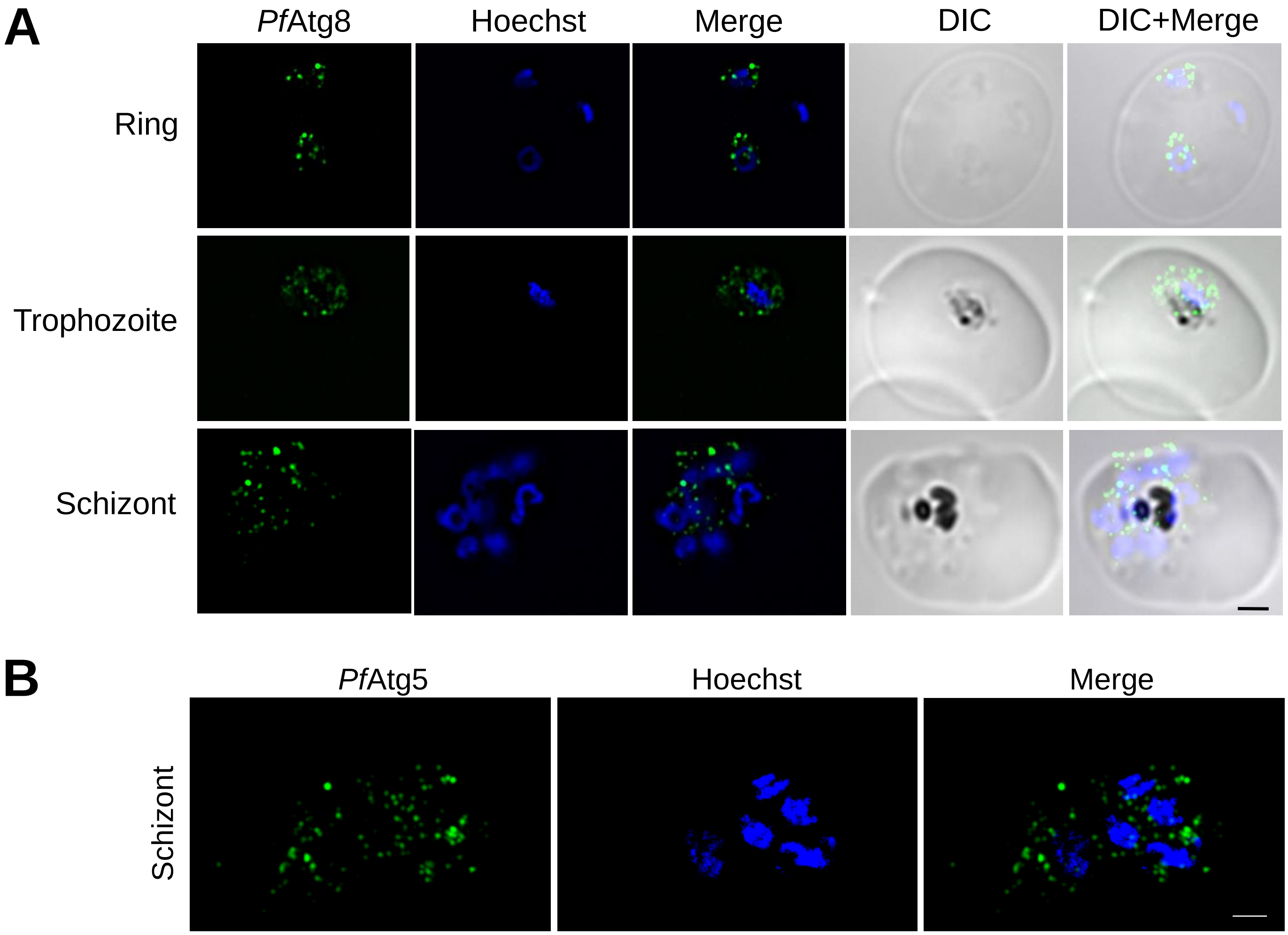

Supplementary Figure S2

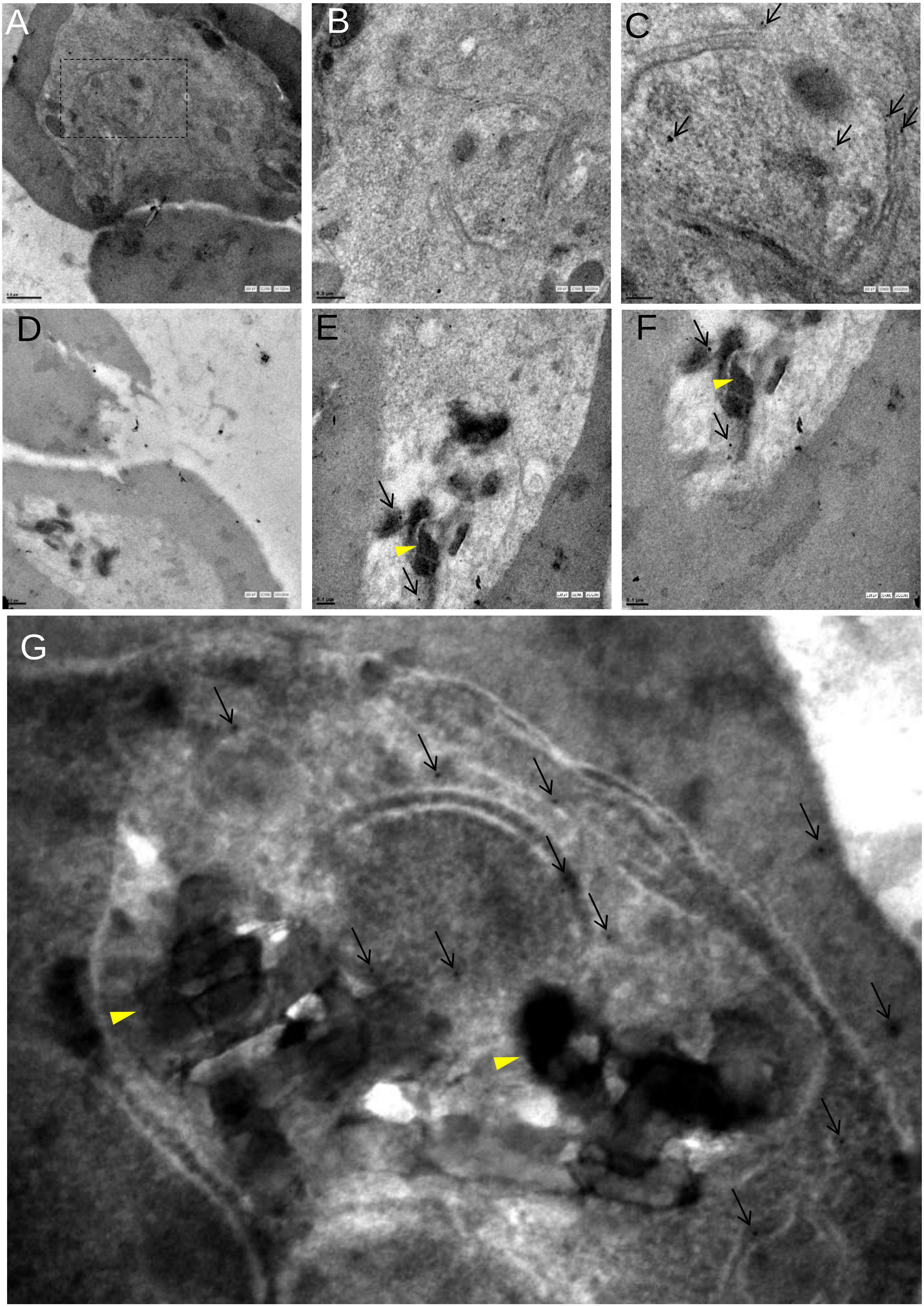

Supplementary Figure S3

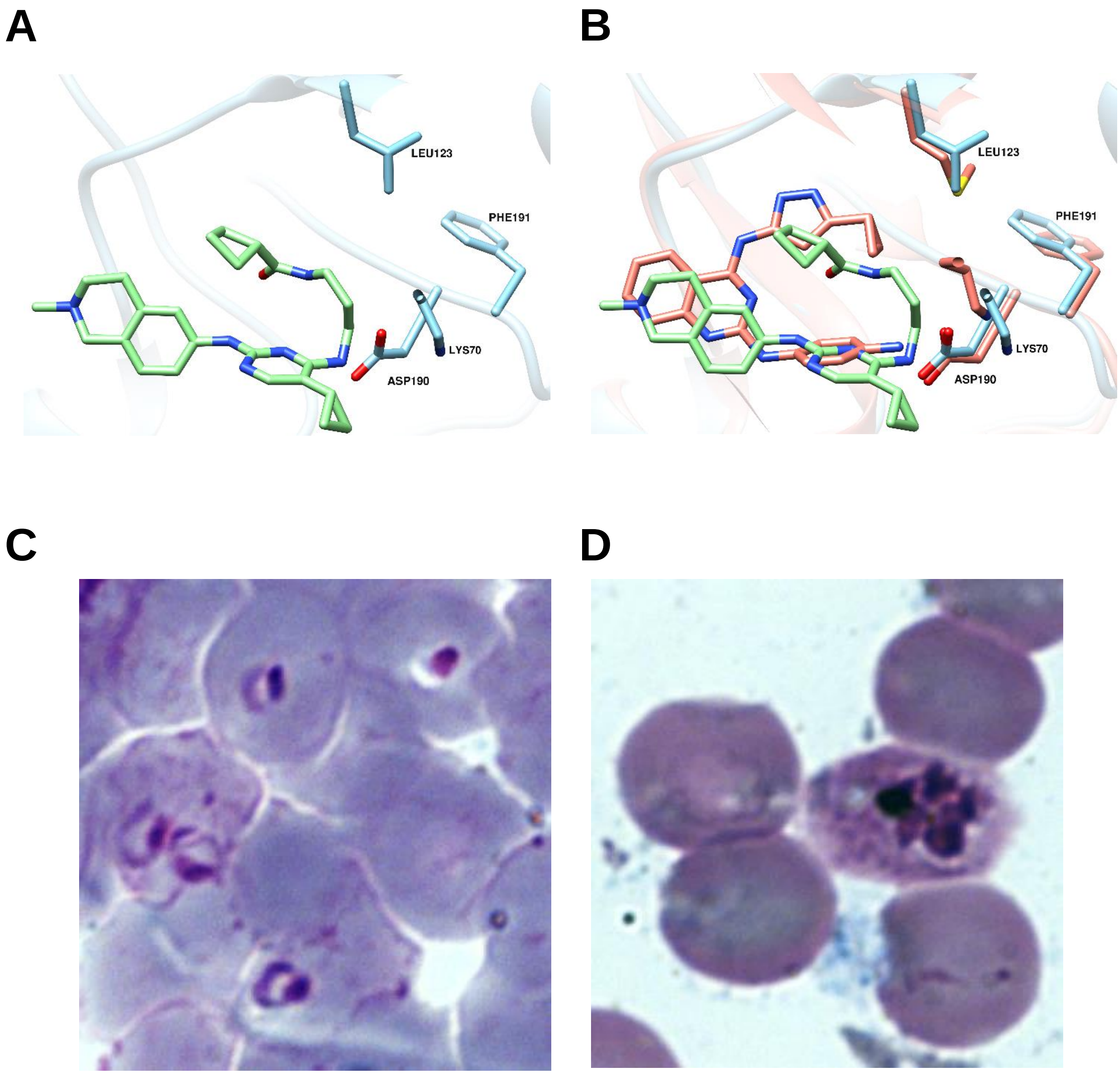

Supplementary Figure S4

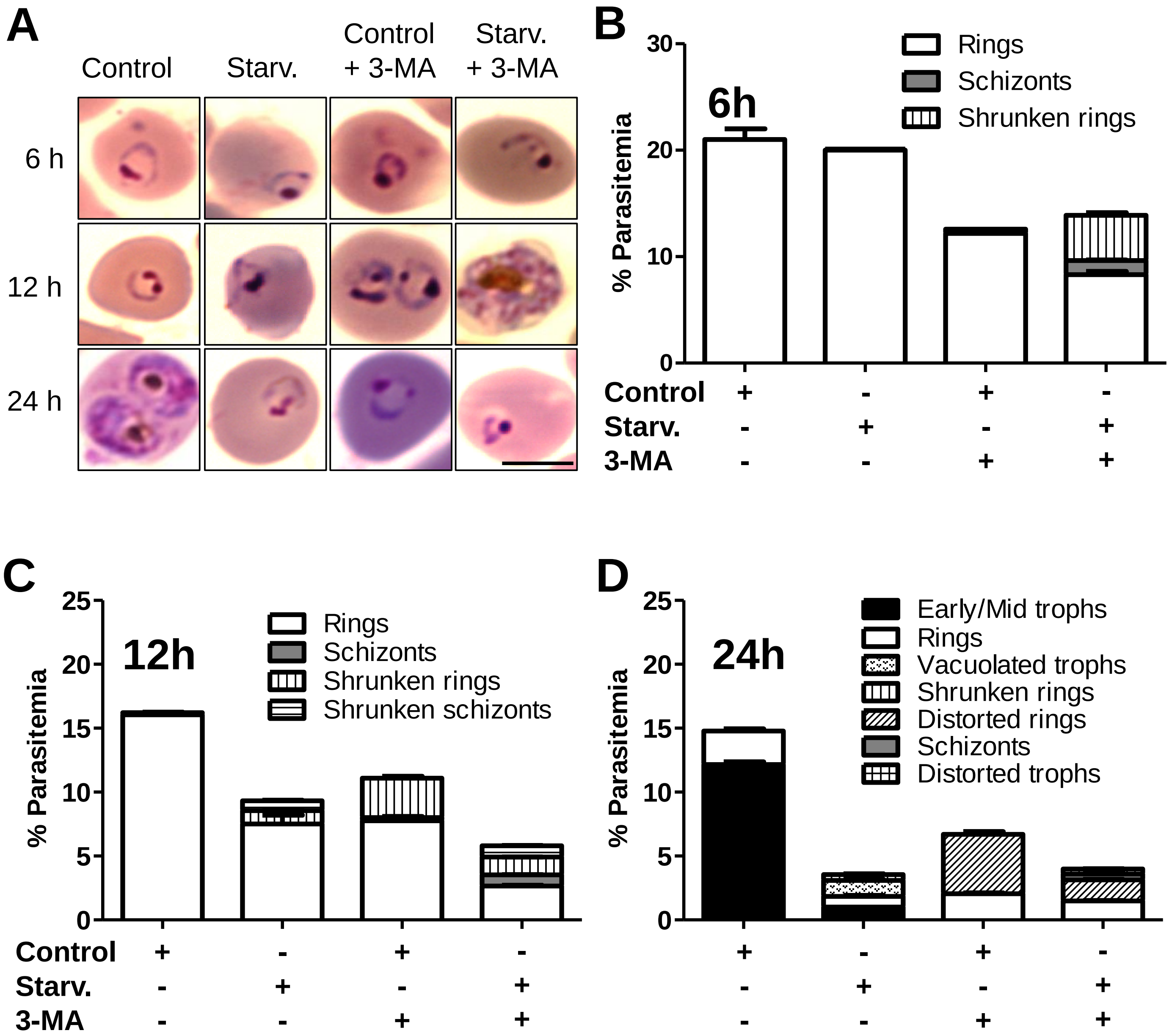

1C

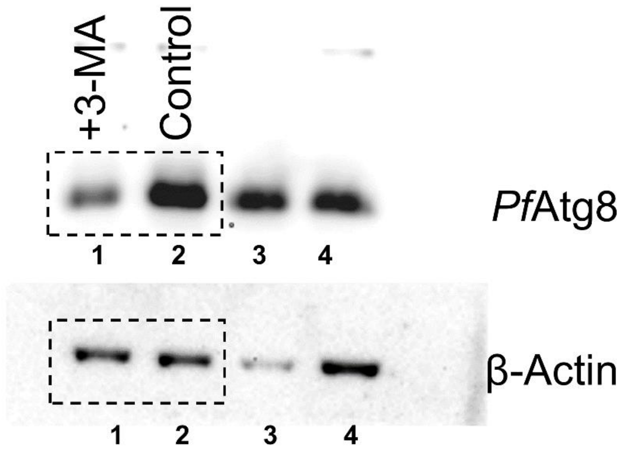
+3-MA
Control
*Pf*Atg8
1 2 3 4
β-Actin
1 2 3 4

2C

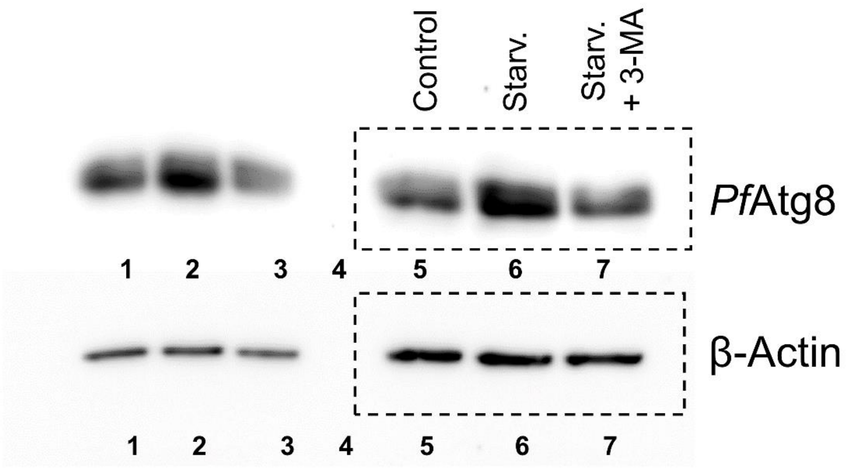
Control
Starv.
Starv. + 3-MA
*Pf*Atg8
1 2 3 4 5 6 7
β-Actin
1 2 3 4 5 6 7

3A

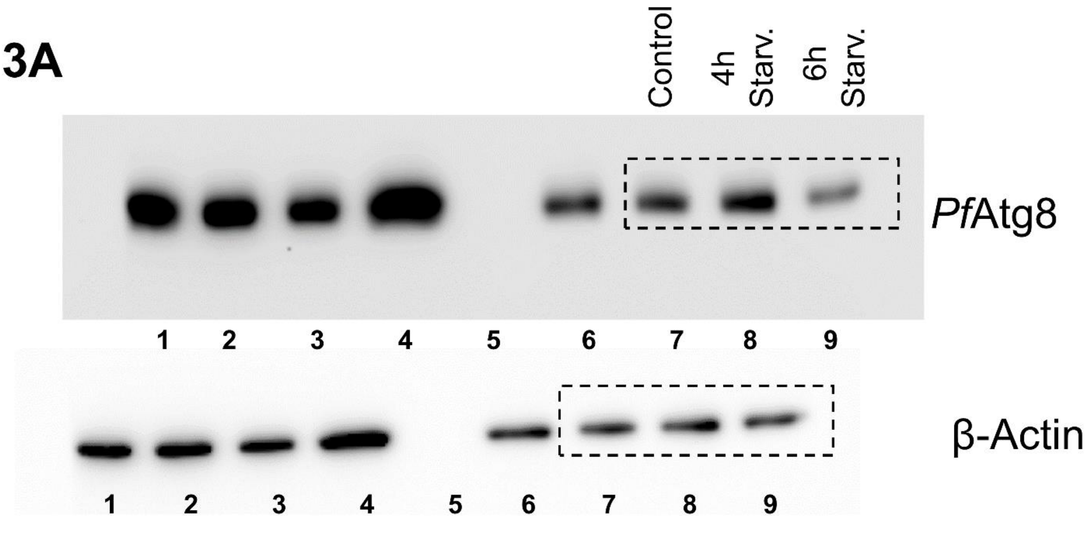
Control
4h Starv.
6h Starv.
*Pf*Atg8
1 2 3 4 5 6 7 8 9
β-Actin
1 2 3 4 5 6 7 8 9
